# Bacterial Lighthouses—Real-Time Detection of *Yersinia enterocolitica* by Quorum Sensing

**DOI:** 10.3390/bios11120517

**Published:** 2021-12-16

**Authors:** Julia Niehues, Christopher McElroy, Alexander Croon, Jan Pietschmann, Martin Frettlöh, Florian Schröper

**Affiliations:** 1 Fraunhofer Institute for Molecular Biology and Applied Ecology IME, Forckenbeckstraße 6, 52074 Aachen, Germany; julia.niehues@ime.fraunhofer.de (J.N.); christopher.mcelroy@ime.fraunhofer.de (C.M.); alexander.croon@ime.fraunhofer.de (A.C.); jan.pietschmann@ime.fraunhofer.de (J.P.); 2 Quh-Lab Lebensmittelsicherheit, Siegener Str. 29, 57080 Siegen, Germany; martin.frettloeh@quh-lab.de

**Keywords:** autoinducer, co-cultivation bioassay, food safety, foodborne pathogens, *N*-acyl homoserine lactones, plasmid-based biosensor, *Yersinia enterocolitica*

## Abstract

Foodborne zoonotic pathogens have a severe impact on food safety. The demand for animal-based food products (meat, milk, and eggs) is increasing, and therefore faster methods are necessary to detect infected animals or contaminated food before products enter the market. However, conventional detection is based on time-consuming microbial cultivation methods. Here, the establishment of a quorum sensing-based method for detection of foodborne pathogens as *Yersinia enterocolitica* in a co-cultivation approach using a bacterial biosensor carrying a special sensor plasmid is described. We combined selective enrichment with the simultaneous detection of pathogens by recording autoinducer-1-induced bioluminescent response of the biosensor. This new approach enables real-time detection with a calculated sensitivity of one initial cell in a sample after 15.3 h of co-cultivation, while higher levels of initial contamination can be detected within less than half of the time. Our new method is substantially faster than conventional microbial cultivation and should be transferrable to other zoonotic foodborne pathogens. As we could demonstrate, quorum sensing is a promising platform for the development of sensitive assays in the area of food quality, safety, and hygiene.

## 1. Introduction

In 1959, the World Health Organization (WHO) defined zoonoses as diseases that are naturally transmittable between humans and other vertebrates [1]. Up to 60% of human infectious diseases are thought to have a zoonotic origin [2]. Due to the growth of the human population, the demand for food products such as meat, milk and eggs, and the intensification of production is increasing. By this, the risk of zoonotic pathogens contaminating food and causing severe health effects is also increasing [3]. Campylobacteriosis was the most common zoonosis reported in 2019 with approximately 221,000 cases in the European Union, followed by salmonellosis (88,000 cases), infections with shiga toxin-producing *Escherichia coli* (STEC) (8000 cases), and yersiniosis (7000 cases) [4]. In 99% of yersiniosis cases, the agent was *Yersinia enterocolitica* [5].

The genus *Yersinia* (family *Enterobacteriaceae*) comprises 19 species, including the human and animal pathogens *Y. enterocolitica*, *Y. pestis*, and *Y. pseudotuberculosis* [6]. *Y. enterocolitica* is a Gram-negative bacillus, with a coccoid to rod-shaped morphology. This psychrophilic species can grow at temperatures within the range 0–45 °C, with optimal growth at 25–28 °C. *Y. enterocolitica* is motile at 25 °C, but loses motility at 37 °C [7,8]. The heterogeneous strains of *Y. enterocolitica* are assigned to six biotypes on the basis of their biochemical features (1A, 1B, and 2–5) and comprise more than 70 serotypes depending on their O-lipopolysaccharide determinants [9,10]. Generally, biotype 1B strains are highly virulent, whereas biotypes 2–5 have comparably low virulence. Strains of biotype 1A are generally non-virulent. Virulence is conferred by the plasmid pVY and virulence-associated chromosomal genes [11]. Nevertheless, some biotype 1A strains cause yersiniosis even though they lack these virulence components [6].

*Y. enterocolitica* is usually transferred to humans via the consumption of contaminated raw or undercooked pork, or by direct contact with contaminated carcasses [12]. Yersiniosis is an acute form of gastroenteritis that often includes symptoms such as fever and intestinal inflammation with watery or occasionally bloody diarrhea [7]. Pigs are the main reservoir, carrying this pathogen in their tonsils, lymphatic tissues, and intestine. As infected pigs are asymptomatic, the bacteria usually were not detected until the infected tissue is exposed during slaughter and processing, allowing the pathogen to spread over the surface of the carcasses [12,13]. Especially due to the devastating health effects, a regular testing for zoonotic pathogens is extremely important. However, rapid and reliable (molecular) detection methods for analysis of meat are not yet available [13].

The conventional detection of *Y. enterocolitica* requires microbial cultivation. An isolation step is necessary, consisting of selective enrichment followed by plating onto selective agar medium. Presumptive colonies are identified by biochemical and pathogenic phenotype. Depending on the choice of enrichment strategy, isolation may take from 48 h up to several weeks [14,15,16]. The conventional method is therefore time-consuming, laborious, and makes it difficult to detect low numbers of pathogens in the presence of abundant background flora also capable of growing on selective media [16].

The polymerase chain reaction (PCR) allows the genotypic characterization of *Y. enterocolitica* by amplifying the virulence-associated genes. Multiplex PCRs have been developed, and more sensitive detection can be achieved by real-time PCR [6,12,13,17,18,19]. PCR is useful for preliminary screening because it enables fast and sensitive analysis, but it requires expensive equipment and skilled personnel. Establishing reliable assays is not trivial since the presence of inhibitors in untreated samples often leads to false-negative results. Additionally, false-positive results may arise due to the detection of dead cells [15]. Immuno-based methods such as the enzyme-linked immunosorbent assay (ELISA) or lateral flow assays for whole cell detection are rare. ELISA formats detecting *Y. enterocolitica* directly are usually serotype-specific and are not well suited for sensitive detection of a bacterial infection [9]. Indirect diagnostic assays were developed to detect antibodies against *Y. enterocolitica* in animal blood and fecal samples [20,21]. However, this only allows the detection of *Y. enterocolitica*-specific antibodies, which only permits a conclusion about (a possible past) yersiniosis. Hence, these methods are not applicable for the detection of an actual bacterial contamination, e.g., in food samples. By this, current commercially available ELISA-based methods are not feasible for efficient analysis and detection of food contamination with *Y. enterocolitica*.

To address the limitations of conventional detection methods and current molecular assays, we developed a new approach for the rapid and sensitive detection of bacteria using quorum sensing (QS) signaling molecules. QS is generally known as a bacterial cell-cell communication process to coordinate gene expression depending on population density. In contrast to low cell density (LCD) populations, at which individual gene expression is favored, the synchronous expression of multiple genes is beneficial at high cell density (HCD) populations. QS-controlled processes include antibiotic production, sporulation, bioluminescence, biofilm formation, and virulence factor secretion. The QS system is based on signaling molecules called autoinducers (AIs), which are produced and secreted by cells. After reaching a local threshold concentration at HCD, the AIs trigger a signal cascade that modulates gene expression [22,23].

The AIs are divided into three major groups: AI-1 (AI-1), AI-2 (AI-2), and peptide-based AIs (AIPs) [24]. AIPs are a heterogeneous group of modified oligopeptides used by Gram-positive bacteria mainly for intraspecies communication [25]. AI-2 is a group of 4,5-dihydroxy-2,3-pentanedione (DPD) derivatives that can rapidly cyclize into furanone counterparts. AI-2 compounds are produced by a wide range of Gram-positive and Gram-negative bacteria and can be used to communicate between cells of the same or different species [26]. AI-1 compounds are *N*-Acyl homoserine lactone-based molecules (AHLs), consisting of a homoserine lactone ring moiety and an acyl side chain, used for intraspecies communication in Gram-negative bacteria [27]. AHL specificity is determined by the length, substitution, and saturation of the acyl side chain [28]. *Y. enterocolitica* produces the AHLs *N*-hexanoyl-L-homoserine lactone (HHL) and *N*-(3-oxohexanoyl)-L-homoserine lactone (OHHL) [29]. Therefore, we focused on the detection of AI-1 in this study.

The best-characterized QS system as a paradigm of intraspecies communication among Gram-negative bacteria is the LuxI/R system of the bioluminescent bacterium *Vibrio fischeri.* The generation of bioluminescence is regulated by the LuxI and LuxR proteins. LuxI is an AHL synthase that produces OHHL. The *luxI* gene is normally expressed at a basal level, resulting in the production of small quantities of OHHL [30]. AHLs are amphipathic molecules that can pass the cell membrane by diffusion [28]. As the cell population grows, the intracellular and extracellular concentration of AHL increases. When it reaches a local threshold concentration, the cytoplasmic LuxR protein binds to OHHL, and the resulting complex functions as a transcriptional activator, inducing the expression of *luxI* and the *lux* operon (*luxCDABEG*). LuxI therefore initiates a positive feedback loop by synthesizing the ligand that binds to LuxR and induces the synthesis of more LuxI. The increase in OHHL levels also triggers the expression of the *lux* operon in adjacent cells [30,31]. The structural genes of the *lux* operon generate bioluminescence via a luciferase-catalyzed oxidation reaction [32].

Within the last decades, the application of bacterial biosensor techniques has advanced the study of AHLs (AI-1) in QS systems and enables a simple method for qualitative and quantitative detection of AHLs [33,34,35]. The bacterial biosensor, which does not produce AHLs by itself, carries a sensor plasmid that encodes the *luxR* gene or the gene of a LuxR homologue, the cognate promoter, as well as a reporter gene for the detection. The presence of a compatible AHL finally induces the expression of the reporter gene whose product is detectable by, for example, bioluminescence [33]. In a previous study, several sensor plasmids (pSB401, pSB403, pSB406, pSB1075) were constructed by linking *luxR* together with the promoter *luxI’*, or homologues as *lasR* and *rhlR* with corresponding promoters, to the *lux* operon (*luxCDABE*) of *Photorhabdus luminescens* [36]. This *lux* operon offers a non-destructive real-time detection as a reporter gene and a higher temperature stability than the *lux* operon of *V. fischeri*, which is relevant for expression in recombinant *E. coli* at 37 °C [36,37]. Additionally, the induction of bioluminescence formation by activating AHLs was demonstrated [36].

In contrast, physico-chemical methods such as GC-MS or LC-MS/MS are frequently used for identification and quantification of AHLs due to their high specificity. However, these devices are highly expensive and need highly trained staff for handling, running, and maintaining the equipment. Furthermore, for method development highly trained staff are required, and sensitivity can vary greatly depending on method parameters, machine settings, and sample preparation. As demonstrated by Bauer and colleagues (2016), GC-MS methods, e.g., showed LODs of just 3.2 to 6.2 µM of single AHLs [38]. Targeted LC–MS/MS methods showed LODs of 0.23 to 1.51 nM [39]. Untargeted LC-MS/MS methods that are capable of detecting and identifying known and unknown AHL entities of a sample showed LODs of 0.6 to 229.6 nM [40]. Whole cell Biosensor approaches, as recently reviewed by Miller and Gilmore (2020), reach higher sensitivities, typically in the low nM and pM ranges [35].

**Figure 1:**
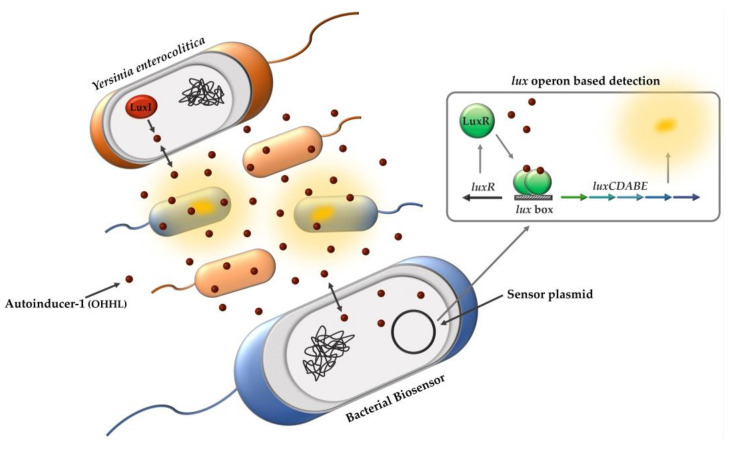
Schematic illustration of whole cell biosensor approach for *Y. enterocolitica* detection based on QS. OHHL produced and secreted by *Y. enterocolitica* induces the *lux* operon in recombinant *E. coli* biosensor resulting in luminescent signal response.

To overcome the limitations of conventional cultivation methods, we established a new whole-cell biosensor approach that enables faster, more sensitive, and more reliable detection of *Y. enterocolitica* based on QS. Therefore, we used the *V. fischeri* LuxI/R system for detection of *Y. enterocolitica* by monitoring the production of its AHLs using a bacterial biosensor carrying a modified sensor plasmid derived from pSB401 [36]. The presence of OHHL in a sample induces the *lux* operon by binding the LuxR protein, inducing bioluminescence and indicating whether the sample is contaminated with *Y. enterocolitica* (Figure 1).

We first investigated the functionality of the biosensor in the presence of synthetic OHHL. We proved the ability to recognize AHLs produced by *Y. enterocolitica* and simultaneously tracked the AHL production of this pathogen via LC-MS/MS analysis for comparison. Finally, biosensor and pathogen were combined in an assay approach to achieve the highest sensitivity possible.

## 2. Materials and Methods

### 2.1. Sensor Plasmid pMA-RQ_luxR_lux

The customized sensor plasmid pMA-RQ_luxR_lux was synthesized by GeneArt (Thermo Fisher Scientific, Waltham, MA, USA). It was based on the sequence of pSB401 [36], which encodes a fusion construct combining the *Vibrio fischeri luxRI*’ and *Photorhabdus luminescens luxCDABE* sequences. The fusion construct was joined to the pMA-RQ backbone, which contains an ampicillin-resistance gene and a ColE1 origin of replication (Figure S1).

### 2.2. Generation of the pMA Biosensor

The pMA-RQ_luxR-lux sensor plasmid was transformed into chemically competent *Escherichia coli* strain BL21 (DE3) cells (New England Biolabs, Frankfurt am Main, Germany) according to the manufacturer’s protocol. Aliquots of 50 µL and 100 µL and the pellet were plated on lysogeny broth (LB) selection plates (10 g L^−1^ tryptone, 5 g L^−1^ yeast extract, 10 g L^−1^ NaCl, 15 g L^−1^ agar; pH 7.4) containing 100 µg mL^−1^ ampicillin. After an overnight incubation at 37 °C, successfully transformed cells were isolated on further selection plates and incubated overnight (37 °C). A colony was then used for preparation of fresh overnight cultures of the biosensor. 

### 2.3. Luminescence Bioassay Procedure

An overnight culture in 5 mL selective LB was prepared with a clone from the master plate and incubated overnight at 37 °C, shaking at 160 rpm. On the next day, 25 mL of selective LB broth was inoculated with the overnight culture at a 1:1000 ratio. The biosensor culture was cultivated at 37 °C, shaking at 160 rpm, until the culture reached OD_600_ = 0.4. The culture was directly used for the luminescence assay. We mixed 20 µL of sample (synthetic AI standard concentrations or sterile culture supernatants) with 180 µL of the biosensor culture in C8 LockWell Lumi white 96-well plates (Thermo Fisher Scientific, Massachusetts, USA). For background measurement, the corresponding buffer or medium was used as blank. Kinetic measurements were carried out for 5 h at 30 °C recording luminescence signals every 20 min using a CLARIOstar plate reader (BMG Labtech, Ortenberg, Germany) with the following set parameters: top optic, 11.0 mm focal height, 10 s measuring interval time, 10 s orbital shaking at 200 rpm before measurement. 

### 2.4. Preparation of Synthetic AI Standard

HHL and OHHL were purchased from Merck (Darmstadt, Germany). Stock solutions with a concentration of 20 mM were prepared in DMSO and stored at −20 °C. On the day of the bioassay, the stock was freshly diluted with sterile phosphate-buffered saline (PBS; 137 µM NaCl, 2.7 mM KCl, 8.1 mM Na_2_HPO_4_, 1.5 mM KH_2_PO_4_; pH 7.4) to concentrations ranging from 0.3 nM up to 200 nM and used as standard concentrations for calibration in the luminescence bioassay.

### 2.5. Cultivation of Y. enterocolitica

*Y. enterocolitica* strain DSM 11503 (biovar 3, serovar 0:9) was obtained from the DSMZ-German Collection of Microorganisms and Cell Cultures (Braunschweig, Germany). Overnight cultures were prepared by inoculating 5 mL tryptic soy broth (TSB, 17 g L^−1^ casein peptone, 3 g L^−1^ soy peptone, 2.5 g L^−1^ d(+) glucose, 5 g L^−1^ NaCl, 2.5 g L^−1^ K_2_HPO_4_; pH 7.3) with 5 µL of a thawed *Y. enterocolitica* glycerol stock. For experimental cultures, selective CIN medium was inoculated with the overnight culture at a ratio of 1:1000. The selective medium was derived from the CIN agar formulation as previously described [41]. Agar and color indicators were omitted from the preparation (17 g L^−1^ gelatin peptone, 1.5 g L^−1^ casein peptone, 1.5 g L^−1^ animal tissue peptone, 2 g L^−1^ yeast extract, 20 g L^−1^ mannitol, 0.5 g L^−1^ sodium deoxycholate, 0.5 g L^−1^ sodium cholate, 1 g L^−1^ sodium chloride, 2 g L^−1^ sodium pyruvate, 0.01 g L^−1^ magnesium sulfate, 0.004 g L^−1^ irgasan, 0.4 g L^−1^ cefsulodin, 0.25 g L^−1^ novobiocin; pH 7.4). All cultures were incubated at 28 °C, with shaking at 160 rpm.

#### 2.5.1. Procedure of Growth Curve Analysis

A growth curve of *Y. enterocolitica* was recorded by inoculating 300 mL selective CIN medium with an overnight culture (1:1000) and taking 12 mL samples at regular intervals, starting at t = 0 and ending at 24 h. The optical density was measured at 600 nm (OD_600_) to determine cell growth. Culture supernatants were generated from 10 mL aliquots (Section 2.5.2), and the cell number was estimated from the OD_600_ on the basis of a *Yersinia*-specific correlation factor determined in the context of this study by plating experiments. An OD_600_ value of 1.0 corresponds to 2.84 · 10^8^ ± 3.9% *Y. enterocolitica* cells mL^−1^. The estimated cell concentration *X* was plotted semi-logarithmically against the cultivation time. The specific growth rate µ was determined by linear regression in the exponential phase. The specific growth rate µ of *Y. enterocolitica* and the doubling time t_D_ was calculated using the following equations [42]:

(1) $$µ=\frac{{\mathrm{lnX}}_{t}\;{-\;\mathrm{lnX}}_{0}}{{t\;-\;t}_{0}}$$

(2) $$t_{D}=\frac{\ln2}{µ}$$

#### 2.5.2. Preparation of Bacterial Culture Supernatants

Sterile culture supernatants were prepared from 10 mL samples taken during the growth curve experiment (Section 2.5.1). Bacterial cells were pelleted by centrifugation (400× *g* for 5 min). The supernatants were filtered using a 0.2 µm syringe sterile filter (Carl Roth, Karlsruhe, Germany) and subsequently stored at −80 °C.

### 2.6. Extraction of AHLs

AHLs were extracted from sterile culture supernatants by liquid-liquid extraction (LLE) in pro-analysis-grade ethyl acetate (Carl Roth, Karlsruhe, Germany) containing 1% (*v*/*v*) analytical grade anhydrous acetic acid (Merck, Darmstadt, Germany). For this, acidified ethyl acetate was added to collected supernatant (1:3 *v*/*v*) and vortexed for 1 min. Afterwards, the organic phase was collected, and the remaining aqueous phase was extracted again as described. In total, five extraction cycles were performed. Pooled organic phases were concentrated by evaporation before drying using a SpeedVac vacuum concentrator (Eppendorf, Hamburg, Germany) for 1 h at RT. For LC-MS/MS analysis, the residue was reconstituted in 200 µL acetonitrile (LC-MS-grade, Carl Roth, Karlsruhe, Germany). To determine the performance and recovery rates achieved using the LLE protocol, we spiked the culture supernatants with 100 ng of OHHL and HHL as additional samples.

### 2.7. Analysis of Extracted AHLs Using LC-MS/MS

AHL extracts were passed through a Claristep 0.2 µm regenerated cellulose filter with an area of 0.7 cm^2^ (Sartorius, Göttingen, Germany) into autosampler vials with inserts. The samples were separated by HPLC on a Curosil-PFP 3 µm 150 × 3.0 mm column (Phenomenex, Aschaffenburg, Germany) using the Agilent 1200 HPLC system with a diode array detector set to 190–400 nm. The mobile phases were (A) degassed ddH_2_O containing 5 mM LiChropur ammonium acetate (Merck), 0.1% (*v/v*) HPLC-grade trifluoroacetic acid (Merck, Darmstadt, Germany), and 0.1% (*v/v*) ACS-grade formic acid (Carl Roth) and (B) ultra-LC-MS/MS-grade acetonitrile (Carl Roth, Karlsruhe, Germany) containing 0.1% (*v/v*) trifluoroacetic acid. We injected 10 µL samples and separated them by gradient flow at a constant flow rate of 0.4 mL min^−1^. The gradient was held at 100% A for 2 min before changing to 100% B over a 12 min linear gradient, followed by a 2 min hold step at 100% B before returning to 100% A over 4 min, followed by a final re-equilibration for 5 min at 100% A. For calibration, stock solutions of OHHL and HHL (2 mg mL^−1^, dissolved in DSMO) were thawed and diluted with acetonitrile to the following concentrations: 0.01, 0.05, 0.1, 0.15, 0.3, 0.5 µg mL^−1^.

AHL extracts were passed through a Claristep 0.2 µm regenerated cellulose filter with an area of 0.7 cm^2^ (Sartorius, Göttingen, Germany) into autosampler vials with inserts. The samples were separated by HPLC on a Curosil-PFP 3 µm 150 × 3.0 mm column (Phenomenex, Aschaffenburg, Germany) using the Agilent 1200 HPLC system with a diode array detector set to 190–400 nm. The mobile phases were (A) degassed ddH_2_O containing 5 mM LiChropur ammonium acetate (Merck), 0.1% (*v/v*) HPLC-grade trifluoroacetic acid (Merck, Darmstadt, Germany), and 0.1% (*v/v*) ACS-grade formic acid (Carl Roth) and (B) ultra-LC-MS/MS-grade acetonitrile (Carl Roth, Karlsruhe, Germany) containing 0.1% (*v/v*) trifluoroacetic acid. We injected 10 µL samples and separated them by gradient flow at a constant flow rate of 0.4 mL min^−1^. The gradient was held at 100% A for 2 min before changing to 100% B over a 12 min linear gradient, followed by a 2 min hold step at 100% B before returning to 100% A over 4 min, followed by a final re-equilibration for 5 min at 100% A. For calibration, stock solutions of OHHL and HHL (2 mg mL^−1^, dissolved in DSMO) were thawed and diluted with acetonitrile to the following concentrations: 0.01, 0.05, 0.1, 0.15, 0.3, 0.5 µg mL^−1^.

The HPLC fractions were analyzed on an API 3000/3200 Q TRAP device (Applied Biosystems, Darmstadt, Germany) equipped with an electro spray ionization probe. Mass scans were carried out in positive ion mode with the following parameters: curtain gas (30 AU), collision gas (medium), ion spray voltage (5500 V), source temperature (550 °C), ion source gas 1 (40), ion source gas 2 (50). AHLs were detected in two multiple reaction monitoring (MRM) experiments, one based upon the selective 102.1 Da lactone ring fragment shown previously to be well suited for AHL detection [43], and the other ion unique to the AHL under investigation. The optimized parameters for each AHL are described in the supplemental methods (Table S1).

### 2.8. Co-Cultivation of Y. enterocolitca and pMA Biosensor

Overnight cultures of the biosensor and *Y. enterocolitica* were prepared as described in Section 2.3 and 2.5. On the next day, 25 mL of CIN medium without cefsulodin, irgasan, or novobiocin was inoculated with the biosensor overnight culture at a ratio of 1:1000, and 20 mL of selective CIN medium was inoculated with the *Yersinia* overnight culture at the same ratio. The biosensor culture was incubated at 37 °C, shaking at 160 rpm, until an OD_600_ reached 0.4, at which point the culture was directly used in the bioassay. The *Yersinia* culture was incubated at 28 °C, shaking at 160 rpm, and was diluted to defined OD_600_ values once the OD_600_ had reached or slightly exceeded 0.05. These defined values were based on cell numbers previously selected for this study. The corresponding OD_600_ was estimated using the specific correlation factor (Section 2.5.1).

Similar to the method described in Section 2.3, 180 µL biosensor culture was mixed with 20 µL culture sample in each well of a C8 LockWell Lumi white 96-well plate (Thermo Fisher Scientific, Waltham, MA, USA). Kinetic measurement was carried out for 20 h at 30 °C, with detection of the luminescence signal every 20 min. The following parameters were set for the top optic measurement: 11.0 mm focal height, 10 s measuring interval, 10 s orbital shaking at 200 rpm before measurement, 0.1 s settling time. Optimized assays were conducted using the bottom optic measurement in a white 96-well microplate with a clear bottom and lid (Berthold Technologies, Bad Wildbad, Germany). Here, the focal height was set to −2.6 mm.

## 3. Results

### 3.1. Validation of pMA Biosensor

**Figure 2:**
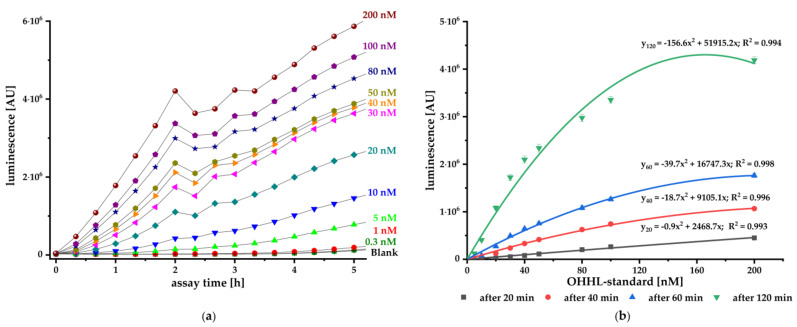
(**a**) Kinetic measurement of luminescence with the pMA biosensor and different standard concentrations of synthetic OHHL AI ranging from 200 nM to 0.3 nM (mean, *n* = 3). (**b**) Averaged calibration curves with corresponding regression functions at different times of luminescence measurement, indicating the relation between the signal generated by the pMA biosensor and OHHL standard concentrations (mean ± SD, *n* = 3).

For evaluation of pMA biosensor functionality, a kinetic luminescence assay for five hours using standard concentrations of the synthetic OHHL AI in combination with pMA biosensor was performed (Figure 2a). PBS without OHHL was used as negative control (blank measurement). On the basis of the kinetic measurement, we were able to establish a correlation of the luminescence signals with different concentration of OHHL after 20, 40, 60, and 120 min by generating calibration curves (Figure 2b). The data were fitted by polynomial regression, and the minimal luminescence signal Lum_min_ was determined on the basis of Equation (3) [44]:

(3) $${\mathrm{Lum}}_{\min}={\;\mathrm{Average}}_{\mathrm{Blank}\;\mathrm{value}}+3\;\cdot {\;\mathrm{SD}}_{\mathrm{Blank}\;\mathrm{values}}$$

By inserting Lum_min_ into corresponding regression function, the lowest detectable concentration of OHHL (LoD, limit of detection) could be calculated. Averaging the corresponding LoDs of three independent experiments, a LoD of 0.50 nM ± 0.29 nM OHHL could be calculated after 20 min of incubation. After further 20 min of incubation, the sensitivity had more than doubled, with a LoD of 0.21 nM ± 0.02 nM OHHL, while extension of the assay time to 60 min or 120 min produced even higher sensitivities of LoD = 0.20 nM ± 0.03 nM or 0.17 nM ± 0.05 nM, respectively.

### 3.2. Detection of AI from Bacterial Cultures

**Figure 3:**
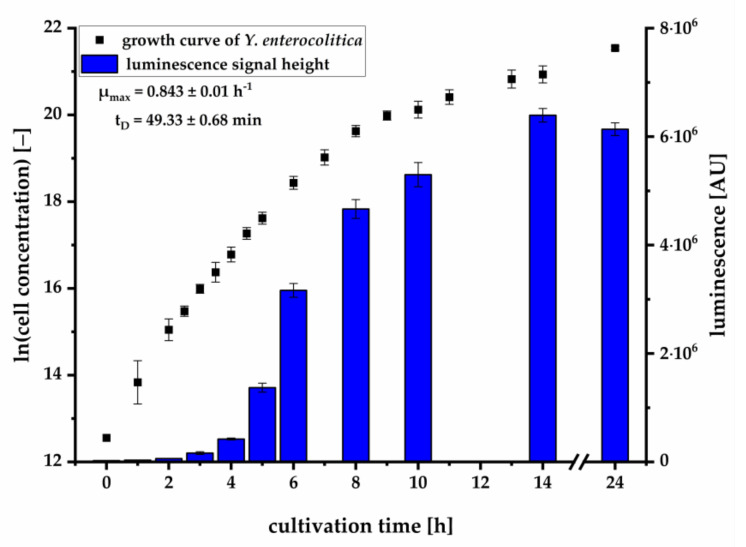
Luminescence signal height after 120 min (mean ± SD, *n* = 3) with the pMA biosensor and culture supernatants plotted against the corresponding growth curve (average of two independent experiments ± SD, *n* = 4, including specific growth rate µ and doubling time t_D_).

On the basis of our results using synthetic OHHL standards, we next focused on the detection of AIs produced by living bacteria. For this, *Y. enterocolitica*, producing OHHL and HHL, was cultivated for 24 h in selective CIN medium. Several samples throughout cultivation were collected for quantifying cell density and analyzing the presence of OHHL by using previously described kinetic luminescence assay. The growth curve of *Y. enterocolitica* is plotted in correlation with the luminescence signals obtained from the sterile filtered supernatant samples after 120 min of kinetic measurement (Figure 3). Although the increase in signal strength lagged slightly behind cell growth, a precise correlation between cell growth and increase in luminescence signal could be detected, demonstrating the potential of luminescence bioassay-based AI-detection. Additionally, a correlation of signal intensity and cell concentration could be detected, indicating the possibility of determining cell concentration with the presented bioassay. 

**Figure 4:**
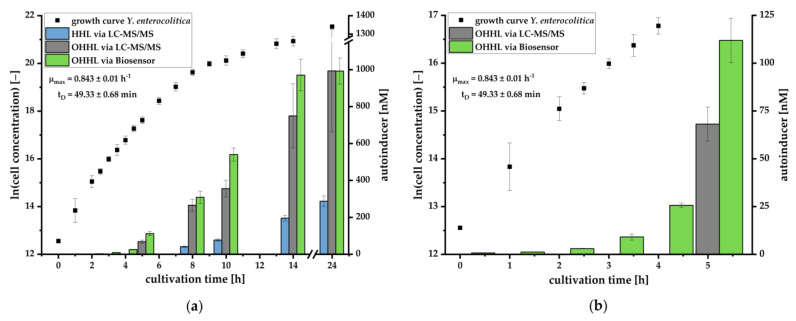
(**a**) Quantification of AI concentrations in culture supernatants of *Y. enterocolitica* by LC-MS/MS (OHHL: gray bars; HHL: blue bars; mean ± SD, *n* = 2) and by pMA biosensor (OHHL: green bars; mean ± SD, *n* = 3 calibration curves at 120 min assay time) in relation to the corresponding growth curve (average of two independent experiments ± SD, *n* = 4; including specific growth rate µ and doubling time t_D_). (**b**) Zoomed section showing cultivation time 0 h to 6 h of cultivation.

As the luminescence signal could be induced by both types of AHLs, OHHL and HHL, the culture supernatants were analyzed regarding the specific AHL concentrations using LC-MS/MS. AHLs were obtained from the supernatants by liquid-liquid extraction and reconstitution in acetonitrile, achieving recoveries of 94.4 ± 9.6% for HHL and 99.2 ± 26.6% for OHHL for all processed samples (*n* = 10). AHL concentrations are compared to the corresponding growth curve in Figure 4a (gray and blue columns). As a result, a 3-6-fold excess of OHHL was detected compared to HHL, leading to the assumption that OHHL is mainly responsible for the luminescence signal observed in Figure 3. Therefore, the AI concentrations were calculated on the basis of OHHL calibration curves after 120 min and compared to the LC-MS/MS-based data in Figure 4a (green columns). A correlation between bacterial growth and the production of AI, which increased substantially during the exponential phase, could be revealed. Here, AI continued to accumulate until the stationary phase.

An AI detection after a cultivation period of 5 h could be achieved using LC-MS/MS. However, the sensitivity was insufficient for detecting lower AI concentrations at earlier cultivation stages. In contrast, the biosensor-based assay reached sufficient sensitivity, and a detection of AHLs in the supernatant from early cultivation stages could be achieved (Figure 4b). 

### 3.3. Co-Cultivation of Y. enterocolitica and pMA Biosensor

The sensitivity of the pMA biosensor-based assay was comparable to our established physico-chemical method based on LC-MS/MS. Although the sensitivity of the bioassay was considerably higher than our established LC-MS/MS protocol, small numbers of *Y. enterocolitica* cells cannot be detected without prior microbiological enrichment, and hence our rapid bioassay requires a short enrichment step. We therefore combined the *Y. enterocolitica* enrichment step with the kinetic measurement of the luminescence signal by co-cultivation. Culture samples containing different densities of proliferating *Y. enterocolitica* cells were added to the pMA biosensor, so both pathogen and biosensor were co-cultivated while the kinetic luminescence measurement was recorded. 

**Figure 5:**
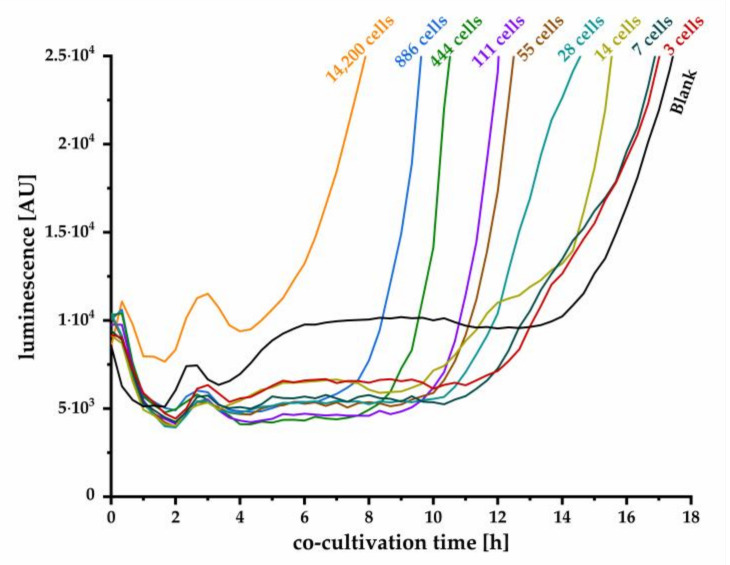
Kinetic measurement of luminescence recorded in intervals of 20 min during the co-cultivation of *Y. enterocolitica* and the pMA biosensor with readout direction from the top (detection plate without cover). Theoretical initial cell numbers (*n* = 3) were estimated using the OD_600_ and specific *Yersinia* correlation factor. The blank signal was obtained from cell medium without *Y. enterocolitica* (*n* = 11).

In Figure 5, the initial signal increase is shown during the co-cultivation, demonstrating the cell number-dependent luminescence response of the pMA biosensor. High initial cell numbers resulted in an early increase of signal. Nevertheless, even the blank sample increased after 13.6 h of co-cultivation, which could be detected in several repetitions of the described experiment. However, the timing of blank-signal increase was not reproducible (data not shown). A distinction of blank samples from samples containing just a small number of cells might not be achievable, resulting in insufficient assay sensitivity. This observation could be explained by evaporation effects when assay plates were used that were not closed using a lid. 

**Figure 6:**
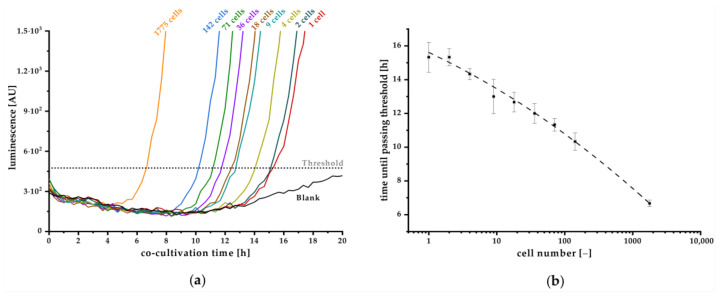
(**a**) Kinetic measurement of luminescence recorded in intervals of 20 min during the co-cultivation of *Y. enterocolitica* and the pMA biosensor according to the optimized co-cultivation approach with readout direction from the bottom (covered detection plate). A threshold was determined on the basis of the averaged blank in relation to the whole assay time and Equation (3). Exceeding the threshold indicates a positive, qualitative signal. Theoretical initial cell numbers (*n* = 3) were estimated using the OD_600_ and specific *Yersinia* correlation factor. The blank signal was obtained from cell medium without *Y. enterocolitica* (*n* = 11). (**b**) Correlation between initial cell number and time until threshold is exceeded fitted with Hill1 equation (R^2^ = 0.998, *n* = 3), enabling a quantitative estimation of the initial cell number.

To counteract, the readout direction was changed to bottom optic, well gaps were filled with water, and the plate was closed using a fitting lid. The optimized method achieved a stable baseline for the blank sample, enabling reliable differentiation from low cell number samples (Figure 6a). A threshold was calculated using Equation (3) on the basis of the averaged blank signal, measured during the entire assay. Exceeding the threshold indicates a positive qualitative detection and the elapsed time before reaching the threshold provides a quantitative estimation of the initial cell number in the sample. As shown in Figure 6b, a correlation between cell number and time until passing the threshold could be detected. By this optimization, the sensitivity could be increased, resulting in a theoretical detection of a single cell *Y. enterocolitica* within a 20 µL sample after co-cultivation for 15.3 h.

## 4. Discussion

We established and demonstrated a concept for rapid and sensitive detection of *Y. enterocolitica* by using QS. Our results proved the feasibility of a real-time detection by monitoring the production of the pathogen’s AHLs using the pMA biosensor. Aiming to overcome the limitations of the conventional detection methods, we successfully adapted a co-cultivation approach to keep microbial enrichment and the simultaneous detection of *Y. enterocolitica* by the pMA biosensor as short and easy as possible.

To validate the pMA biosensor, we carried out kinetic luminescence assays using different standard concentrations of synthetic AI. The biosensor responded concentration-dependent to the authentic AHL and provides reliable and sensitive signals from 20 min onwards (Figure 2). It is particularly suitable for fast detection of small amounts OHHL with a sensitivity ranging from 0.50 nM up to 0.17 nM (LoD). This biosensor is at least as sensitive as the published constructs (pSB401, pSB403, pSB406, and pSB1075) with sensitivity to different AHLs in the pM and nM range [36].

We could further demonstrate that the pMA biosensor is able to respond to AHLs in culture supernatants of *Y. enterocolitica* (Figure 3) without being suppressed by compounds of selective media. The increase of the luminescence signal in relation to the growth of the pathogen demonstrates the characteristic accumulation of AHLs during cell growth. To investigate which AHL is produced during cell growth and is mainly responsible for induction of the *lux* cassette, we analyzed culture supernatants using LC-MS/MS. Here, we successfully verified the presence and production of OHHL and HHL during growth of *Y. enterocolitica*. OHHL is the cognate AI of LuxR and activates the *lux* gene expression most effectively [36]. HHL differs from OHHL by the absence of a carbonyl-group at the C3-atom in the side chain. This OHHL analogue binds with less efficiency to LuxR and induces the *lux* mechanism with less activity as OHHL. A previous study demonstrated that HHL inhibited the binding of OHHL to LuxR by about 10% when molar ratio was 1:1 of OHHL and HHL [45]. Our study revealed that *Y. enterocolitica* produced 3-6 fold less HHL than OHHL. Thus, an inhibition effect is negligible, confirming that the *lux* mechanism is primarily induced by OHHL. This condition allows for quantification of OHHL in culture supernatants by the pMA biosensor. Determined concentrations correlated in great manner with concentrations quantified by LC-MS/MS (Figure 4). The quantification by the pMA biosensor revealed a higher sensitivity than our LC-MS/MS-based analytical method, which by itself revealed a higher sensitivity than published methods demonstrating the ability for *Y. enterocolitica* detection in early stages of cultivation (compare [40]). Further research is necessary to determine whether and to what extent other AHLs may interact with the *lux* promoter. To generate data to the specificity or selectivity of the analytical procedure, we would need to investigate the selectivity of the selective medium and to determine which other bacteria could also produce OHHL and induce luminescent responses. This is especially important in terms of determining how specific our bacterial sensor is and what kind of selective enrichment steps need to be employed.

To date, several approaches have been developed using bacterial biosensors to detect and quantitatively estimate different kinds of bacteria on the basis of their secreted autoinducers [35]. However, sensitivity was not high enough to detect very low cell numbers. Hence, by applying the pMA biosensor in a co-cultivation approach, we could establish a real-time detection of *Y. enterocolitica* for the first time by monitoring produced OHHL. We further demonstrated that even single cell detection and reliable quantification is possible. Although the detection of small cell number requires several hours of co-cultivation, the presented method is considerably faster than classic microbiological cultivation techniques. The assay set up was successfully optimized, resulting in a stable blank signal throughout relevant duration of cultivation, facilitating the determination of a threshold value that defines the minimal luminescence signal, confirming a positive detection. With this approach, we achieved high sensitivity for detection of one initial cell in a sample after 15.3 h of co-cultivation. Hence, monitoring the luminescence signal and the time to exceed threshold also enables quantitative estimation of the initial cell concentration on the basis of the calibration curve plotted in Figure 6b. Here, the detection limit of one cell strongly depends on the reproducible low blank signal as realized in our improved experimental approach in combination with a reliable correlation factor between OD_600_ and cells per milliliter. The quantitative detection range is limited by high cell numbers, where autoinducer concentration is already high enough to induce expression of the *lux* operon. In this case, expression will start instantly, and strong luminescence signal increase can be observed within few minutes, as can be concluded from kinetics in Figure 2. Our established method is more time-effective than the conventional microbiological detection methods and ensures the detection of living cells as the release of AHLs is an active process. For a specific detection of *Y. enterocolitica*, further optimization has to be done, especially excluding luminescence signals induced by AIs from other bacteria. It is known that OHHL and similar AHLs that also might stimulate the *lux* expression cassette in the pMA biosensor are produced by a huge number of *Enterobacteria*. Therefore, a short pre-enrichment incubation step (e.g., 3 h) in CIN selection media might be beneficial to reduce the number of other bacteria and to mainly enrich *Y. enterocolitica*. However, CIN medium is not 100% selective to *Y. enterocolitica*, and therefore additional steps such as, e.g., an alkali treatment, could be necessary to increase selectivity [46,47]. Hence, further experiments have to be performed to reveal the specificity of our here presented assay when using real food samples. Nevertheless, our approach is well suited for fast an early detection of bacterial contamination and could be used in food security or veterinary applications. Further research will focus on the adaption of the luminescence cassette to specifically detect further AI molecules enabling fast detection of other pathogenic bacteria such as *Salmonella*, *Campylobacter*, or *Legionella*. Exchanging the luminescence gene by fluorescence cassettes expressing different fluorophores might additionally enable multiplex detection of different bacteria in a single sample.

## Data Availability

The datasets generated in this study are available from the corresponding author on request.

## Supplementary Materials

Supplementary materials can be found at https://www.mdpi.com/article/10.3390/bios11120517/s1. Figure S1. Plasmid map of sensor plasmid pMA-RQ_luxR_lux. It is based on the sequence of pSB401 [36], encoding a fusion construct combining the *Vibrio fischeri luxRI’* and *Photorhabdus luminescens luxCDABE* sequences. The fusion construct was synthesized and joined to the pMA-RQ backbone by GeneArt. The backbone carries an ampicillin resistance gene and a ColE1 origin of replication. Table S1. Optimized parameters for detection of N-hexanoyl- and N-(3-oxohexanoyl)-L-homoserine lactone using LC–MS/MS.
