# Supplementary material for: Bacterial Lighthouses—Real-Time Detection of Yersinia enterocolitica by Quorum Sensing
Source: Biosensors (Basel). 2021 Dec 16;11(12):517. doi: 10.3390/bios11120517 (PMC8699262; doi:10.3390/bios11120517)
Supplement: Supplementary file 1 [file biosensors-11-00517-s001.zip › biosensors-1474265-supplementary.pdf]

biosensors

MDPI

Supplementary Materials

Bacterial Lighthouses—Real-Time Detection of *Yersinia enterocolitica* by Quorum Sensing

Julia Niehues [1], Christopher McElroy [1], Alexander Croon [1], Jan Pietschmann [1], Martin Frettlöh [2] and Florian Schröper [1,*]

[1] Fraunhofer Institute for Molecular Biology and Applied Ecology IME, Forckenbeckstraße 6, 52074 Aachen, Germany; julia.niehues@ime.fraunhofer.de (J.N.); christopher.mcelroy@ime.fraunhofer.de (C.M.); alexander.croon@ime.fraunhofer.de (A.C.); jan.pietschmann@ime.fraunhofer.de (J.P.)
[2] Quh-Lab Lebensmittelsicherheit, Siegener Str. 29, 57080 Siegen, Germany; martin.frettloeh@quh-lab.de
* Correspondence: florian.schroeper@ime.fraunhofer.de; Tel.: +49-(0)241-6085-13012

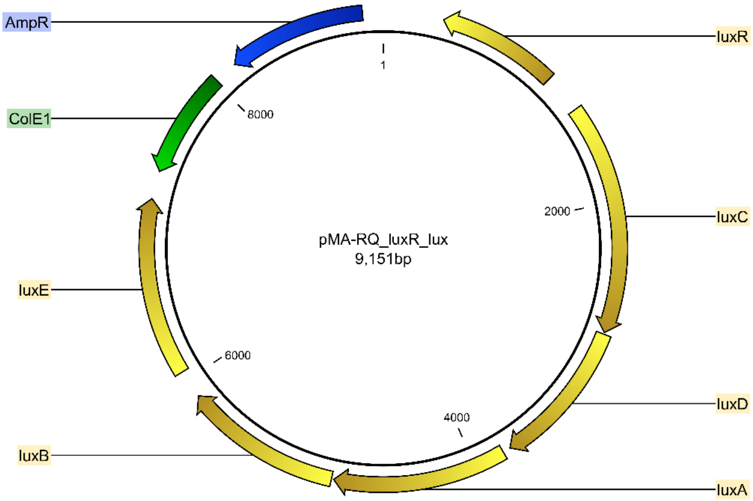

Figure S1. Plasmid map of sensor plasmid pMA-RQ_luxR_lux. It is based on the sequence of pSB401 [36], encoding a fusion construct combining the *Vibrio fischeri luxRI'* and *Photorhabdus luminescens luxCDABE* sequences. The fusion construct was synthesized and joined to the pMA-RQ backbone by GeneArt. The backbone carries an ampicillin-resistance gene and a ColE1 origin of replication.

Table S1. Optimized parameters for detection of *N*-hexanoyl- and *N*-(3-oxohexanoyl)-L-homoserine lactone using LC-MS/MS.

ID	Q1 [Da]	Q3 [Da]	Dwell Time [ms]	DP [V]	EP [V]	CEP [V]	CE [V]	CXP [V]
N-hexanoyl-L-homoserine lactone	200.1	102.1	200	30.0	6.90	12.189	13.670	2.00
N-hexanoyl-L-homoserine lactone	200.1	182.1	200	30.0	6.90	12.189	13.670	2.00
N-(3-oxohexanoyl)-L-homoserine lactone	214.1	102.1	200	26.8	9.18	12.796	12.300	5.39
N-(3-oxohexanoyl)-L-homoserine lactone	214.1	168.1	200	26.8	9.18	12.796	12.300	5.39
